# A cross-sectional study of the associations between intuitive eating and maladaptive eating behaviors in Brazilian University students

**DOI:** 10.1007/s40519-026-01888-8

**Published:** 2026-07-14

**Authors:** Andressa Yavorivski, Ana Corrêa Ruiz, Rui Poínhos, Ana Maria Pandolfo Feoli

**Affiliations:** 1 School of Health and Life Sciences, Pontifical Catholic University of Rio Grande do Sul, Porto Alegre, Brazil; 2 Faculty of Nutrition and Food Sciences, University of Porto, Porto, Portugal; 3 Department of Biology and Environment, School of Life and Environmental Sciences, University of Trás-os-Montes e Alto Douro, Vila Real, Portugal; 4 Postgraduate Program in Psychology, Pontifical Catholic University of Rio Grande do Sul, Porto Alegre, Brazil

**Keywords:** Intuitive eating, Eating behavior, Uncontrolled eating, Emotional eating, Disordered eating

## Abstract

**Purpose:**

To investigate the relationships between Intuitive Eating and maladaptive eating behaviors among Brazilian university students.

**Methods:**

A cross-sectional quantitative study was conducted among technical, undergraduate, and postgraduate students aged 18 years or above, between 2020 and 2021. Participants completed an online survey comprising sociodemographic questions, the Intuitive Eating Scale-2 (IES-2), and the Three-Factor Eating Questionnaire-Revised 21 (TFEQ-R21), both validated for the Brazilian population. Spearman’s correlation and MANCOVA tests were used to assess associations between study variables.

**Results:**

The study included 1,237 participants (82.6% females) with a mean age of 26.3 (SD = 8.0) years. Intuitive Eating and its domains were negatively associated with Cognitive Restraint, Emotional Eating and Uncontrolled Eating. In the MANCOVA model, the Unconditional Permission to Eat subscale (IES-2) explained a large proportion of variance in Cognitive Restraint (η2p = .314), and the Eating for Physical Rather than Emotional Reasons subscale (IES-2) explained a large proportion of variance in Emotional Eating (η2p = .587).

**Conclusion:**

Intuitive eating and its domains were negatively associated with maladaptive eating behaviors among Brazilian university students, particularly Emotional Eating and Cognitive Restraint, with Eating for Physical Rather than Emotional Reasons and Unconditional Permission to Eat showing the strongest relationships.

**Level of evidence:**

Level 4—Cross-sectional study.

## Introduction

Intuitive Eating, pioneered by Evelyn Tribole and Elise Resch in 1995, emphasizes a compassionate and self-care approach to eating, prioritizing respect for all body types. Unlike conventional dieting, it promotes attunement to internal body signals by removing restrictive rules imposed by diet culture [31, 32]. The Intuitive Eating Scale-2 (IES-2) assesses four domains: Unconditional Permission to Eat (allowing oneself to eat without restriction), Reliance on Hunger and Satiety Cues (trust in internal physiological signals), Eating for Physical Rather than Emotional Reasons (eating in response to physical rather than emotional cues), and Body-Food Choice Congruence (aligning food choices with bodily needs) [33]. Intuitive eating has been consistently associated with higher psychosocial well-being, lower BMI, fewer disordered eating behaviors, and greater body appreciation [23].

In contrast, the Three-Factor Eating Questionnaire (TFEQ-R21) assesses eating behaviors related to weight regulation and eating disorders [14]. It comprises three domains: Cognitive Restraint, defined as the intentional restriction of food intake to control weight [2, 12], Uncontrolled Eating, characterized by a tendency to lose control over eating in response to hunger or external cues [12], and Emotional Eating, which refers to overeating in response to negative emotions or stress [35]. These behaviors are considered maladaptive due to their association with adverse outcomes, including weight gain, eating disorders, and psychological distress [10, 29, 36].

The transition to higher education is a critical period marked by behavioral and psychological changes that increase vulnerability to disordered eating [21]. High rates of mental health difficulties have been documented among university students globally and in Brazil, including anxiety, discouragement, and eating-related concerns [5, 20]. Additionally, a substantial proportion of Brazilian students engage in restrictive dieting [1, 25], a pattern associated with maladaptive eating behaviors.

These challenges may have been further intensified during the COVID-19 pandemic due to social isolation and disruptions in daily routines [8]. Emerging evidence indicates a negative relationship between Intuitive Eating and maladaptive eating behaviors [28]. However, the extent to which specific domains of Intuitive Eating are differentially associated with these behaviors remains unclear, particularly in Brazilian populations.

Given its emphasis on internal regulation rather than external control, Intuitive Eating may represent a promising framework for reducing maladaptive eating behaviors and preventing eating disorders. Clarifying these relationships is particularly relevant for university students, a population at elevated risk [23].

Therefore, this study aims to investigate the relationships between Intuitive Eating and maladaptive eating behaviors among Brazilian university students, considering the influence of sex, age, and BMI. We hypothesize that Unconditional Permission to Eat and Eating for Physical Rather than Emotional Reasons will show the strongest associations with Cognitive Restraint, Uncontrolled Eating, and Emotional Eating.

## Methods

### Participants and procedure

The present cross-sectional quantitative study is based on the database of the original project “Relationship between Intuitive Eating and Nutritional Status of Brazilian University Students”, conducted among technical, undergraduate and postgraduate students from Brazil, between September 2020 and September 2021. Participants were recruited using a non-probabilistic convenience sampling strategy through digital platforms (Instagram, Facebook, WhatsApp, ResearchGate) and institutional email lists. Recruitment posts were disseminated using a standardized invitation text targeting Brazilian university students aged ≥ 18 years. Although no algorithmic targeting criteria were applied, efforts were made to maximize sample heterogeneity by distributing invitations across multiple platforms with distinct audience profiles (academic networks, general social media, and institutional mailing lists). Eligibility criteria included: (1) being enrolled in a Brazilian university, and (2) age ≥ 18 years. No restrictions regarding sex, field of study, or geographic region were imposed.

A Free and Informed Consent Form (ICF) providing information about the purpose and procedures of the study was presented, and after accepting it, participants had access to the anonymous online survey in Qualtrics software, version 75 (Qualtrics, Provo, UT, 2020). In the original study, the exclusion criteria were being currently pregnant, unable to participate freely and informed, or completing only sociodemographic data, resulting in 1,246 participants. For the present analysis, eight participants without BMI data and one without age data were excluded, resulting in 1,237 participants. The original study was approved by the Research Ethics Committee of the Pontifical Catholic University of Rio Grande do Sul (PUCRS), the CAAE number is 12444019.8.0000.5336, as stated on Plataforma Brasil. All participants, before starting the research, read and agreed to the ICF, which contains general information about the project, the objective and possible benefits and discomforts. The authors of the present study signed a Data Use Commitment Term.

### Measures

The questionnaire consisted of demographic (i.e. age, sex, socioeconomic status, education level, course), self-reported mental disorder and/or eating disorder, other health closed options questions, the Intuitive Eating Scale-2 (IES-2), the Three-Factor Eating Questionnaire-Revised 21 (TFEQ-R21)and self-reported weight and height. Body mass index (BMI, kg/m^2^) was calculated and then classified according to the World Health Organization.

### Intuitive Eating Scale (IES-2)

Intuitive Eating was assessed using the Intuitive Eating Scale-2 (IES-2) [32], a 23-item instrument comprising four subscales: Unconditional Permission to Eat, Eating for Physical Rather than Emotional Reasons, Reliance on Hunger and Satiety Cues, and Body-Food Choice Congruence. Responses are rated on a 5-point Likert scale, and scores are calculated as the mean of items, with higher scores indicating greater intuitive eating. The IES-2 was translated and validated for the Brazilian population [17].

### Three Factor Eating Questionnaire-Revised 21 (TFEQ-R21)

Eating behaviors were assessed using the Three-Factor Eating Questionnaire-Revised 21 (TFEQ-R21) [14], a 21-item instrument that evaluates three domains: Cognitive Restraint, Emotional Eating, and Uncontrolled Eating. Items are rated on Likert scales, and domain scores are transformed to a 0 to 100 scale, with higher scores indicating greater expression of each behavior. The instrument was translated and validated for the Brazilian population [18, 27].

### Data analysis

Statistical analysis was conducted using JASP 0.18.1. Categorical variables were represented as counts (n) and percentages (%), while continuous variables were expressed as means and standard deviations (SD). The normality of the data was assessed with the coefficients of skewness and kurtosis. To assess potential differences in means between the groups of men and women, Welch's t-tests were conducted. Spearman’s correlations (rs) were then employed to examine the relationships between variables. Due to positive skewness and kurtosis (values > 1), BMI and age were log-transformed before a MANCOVA model was conducted. MANCOVA's effect sizes were quantified using partial eta-squared (η^2^p). The interpretation of effect sizes was based on the qualitative definitions originally proposed by Cohen [16]. Spearman’s correlation coefficients were interpreted as small (weak) when |rs| < 0.2, medium (moderate) when 0.2 ≤ |rs| < 0.4, and large (strong) when |rs| ≥ 0.4. Partial eta-squared values were interpreted as small when ηp^2^ < 0.035, medium when 0.035 ≤ ηp^2^ < 0.100, and large when ηp^2^ ≥ 0.100. Statistical significance was defined as p < 0.05 for all analyses.

## Results

### Participants characteristics

The total sample consisted of 1,237 students, 82.6% of whom were female, with a mean age of 26.3 (SD = 8.0) years. The majority were undergraduates (74.3%) and had normal weight (57.8%). Half of the sample was from the southern region (51.3%). Other sociodemographic characteristics and the IES-2 and TFEQ-R21 scales scores for the total sample and male and female subgroups are available in Table 1.

**Table 1:** Sociodemographic characteristics, IES-2 and TFEQ-R21 scores of total sample and by sex

Characteristic	Total (n = 1237)	Women (n = 1022)	Men (n = 215)
	Mean ± SD or n (%)		
Age (years)	26.3 ± 8.0	26.3 ± 8.0	26.1 ± 8.1
Sex			
Female	1022 (82.6%)		
Male	215 (17.3%)		
BMI			
Underweight (< 18.5 kg/m^2^)	77 (6.2%)	73 (7.1%)	4 (1.8%)
Normal weight ([18.5; 25] kg/m^2^)	716 (57.8%)	602 (58.9%)	114 (53.0%)
Overweight ([25; 30] kg/m^2^)	303 (24.4%)	236 (23.0%)	67 (31.2%)
Obesity I ([30; 35] kg/m^2^)	97 (7.8%)	74 (7.2%)	23 (10.6%)
Obesity II ([35; 40] kg/m^2^)	44 (3.5%)	37 (3.6%)	7 (3.2%)
Region of Brazil			
North	80 (6.4%)	62 (6.1%)	18 (8.3%)
North East	163 (13.1%)	125 (12.2%)	38 (17.6%)
Midwest	54 (4.3%)	39 (3.8%)	15 (6.9%)
Southeast	305 (24.6%)	265 (25.9%)	40 (18.6%)
South	635 (51.3%)	531 (51.9%)	104 (48.3%)
Marital status			
Single	954 (77.1%)	778 (76.1%)	176 (81.8%)
Married	143 (11.5%)	121 (11.4%)	22 (10.2%)
Partnered	139 (11.2%)	122 (11.9%)	17 (7.9%)
Widowed	1 (0.1%)	1 (0.1%)	0
Education status (in progress)			
Technical	19 (1.5%)	9 (0.8%)	10 (4.6%)
Graduation	920 (74.3%)	764 (74.7%)	156 (72.5%)
Postgraduate	298 (24%)	249 (24.3%)	49 (22.7%)
Occupation			
Full-time student	513 (41.4%)	418 (40.0%)	95 (44.1%)
Student and intern	231 (18.6%)	199 (19.4%)	32 (14.8%)
Student and worker	493 (39.8%)	405 (39.6%)	88 (40.9%)
Confinement situation			
Home	64 (5.1%)	44 (4.3%)	20 (9.3%)
Confined at home, except for occasional trips (e.g. shopping, supporting family members)	725 (58.6%)	605 (59.1%)	120 (55.8%)
Regular mobility outside home (e.g. essential professions)	385 (31.1%)	321 (31.4%)	64 (29.7%)
Total mobility, normal life, as I don’t think I have to be confined	63 (5.0%)	52 (5.0%)	11 (5.1%)
Type of housing			
Apartment without balcony and/or terrace	225 (18.1%)	185 (18.1%)	40 (18.6%)
Apartment with balcony and/or terrace	252 (20.3%)	205 (20%)	47 (21.8%)
House without outdoor space (backyard)	68 (5.4%)	58 (5.6%)	10 (4.6%)
House with outdoor space (backyard)	682 (55.1%)	568 (55.5%)	114 (53%)
Other	10 (0.8%)	6 (0.5%)	4 (1.8%)
Lives with			
Alone	144 (11.6%)	113 (11%)	31 (14.4%)
Family	850 (68.7%)	697 (68.2%)	153 (71.1%)
Friends	44 (3.5%)	38 (3.7%)	6 (2.7%)
Student housing	3 (0.2%)	2 (0.1%)	1 (0.4%)
Partner	196 (15.8%)	172 (16.8%)	24 (11.1%)
Income			
No income	19 (1.5%)	16 (1.5%)	3 (1.3%)
One minimum wage^a^	69 (5.5%)	56 (5.4%)	13 (6.0%)
One to three minimum wages	357 (28.8%)	301 (29.4%)	56 (26.0%)
Three to six minimum wages	361 (29.1%)	305 (29.8%)	56 (26.0%)
Six to nine minimum wages	163 (13.1%)	131 (12.8%)	32 (14.8%)
Nine to twelve minimum wages	107 (8.6%)	89 (8.7%)	18 (8.3%)
Twelve to fifteen minimum wages	61 (4.9%)	47 (4.5%)	14 (6.5%)
More than 15 minimum wages	100 (8.0%)	77 (7.5%)	23 (10.6%)
Food allergy or intolerance			
Yes	270 (21.8%)	232 (22.7%)	38 (17.6%)
No	967 (78.0%)	790 (77.2%)	177 (82.3%)
Chronic non-communicable disease			
Yes	204 (16.4%)	172 (16.8%)	32 (14.8%)
No	1033 (83.5%)	850 (83.1%)	183 (85.1%)
Endocrine nutritional metabolic disease			
Diabetes	7 (0.5%)	6 (0.5%)	1 (0.4%)
Hypertension	10 (0.8%)	6 (0.5%)	4 (1.8%)
Dyslipidemia	1 (0.1%)	1 (0.1%)	0
Hypothyroidism	26 (2.1%)	24 (2.3%)	2 (0.9%)
Hyperthyroidism	3 (0.2%)	3 (0.2%)	0
Kidney disease	3 (0.2%)	3 (0.2%)	0
Ovarian dysfunction	16 (1.2%)	16 (1.5%)	0
Obesity	16 (1.2%)	14 (1.3%)	2 (0.9%)
Other	70 (5.6%)	58 (5.6%)	12 (5.5%)
No	1085 (87.7%)	891 (87.1%)	194 (90.2%)
Eating Disorder			
Anorexia Nervosa	5 (0.4%)	5 (0.5%)	0
Bulimia Nervosa	19 (1.5%)	19 (0.4%)	0
Binge Eating Disorder	58 (4.6%)	55 (1.8%)	3 (1.3%)
Eating Disorder Not Otherwise Specified	19 (1.5%)	18 (5.3%)	1 (0.4%)
No	1136 (91.3%)	925 (90.5%)	211 (98.1%)
Mental Disorder			
Depression	80 (6.4%)	72 (7.0%)	8 (3.7%)
Anxiety	265 (21.4%)	231 (22.6%)	34 (15.8%)
Alcohol or Substance Use Disorder	2 (0.1%)	0	2 (0.9%)
Obsessive Compulsive Disorder	11 (0.8%)	9 (0.8%)	2 (0.9%)
Bipolar Disorder	16 (1.2%)	13 (1.2%)	3 (1.3%)
Borderline Personality Disorder	5 (0.4%)	5 (0.4%)	0
Post-traumatic Stress Disorder	4 (0.3%)	4 (0.3%)	0
Attention Deficit Hyperactivity Disorder (ADHD)	17 (1.3%)	12 (1.1%)	5 (2.3%)
Other	6 (0.4%)	6 (0.5%)	0
No	831 (67.2%)	670 (65.5%)	161 (74.8%)
Intuitive Eating Scale			
Total	3.36 ± 0.67	3.33 ± 0.68	3.49^*^ ± 0.57
Unconditional permission to eat	3.58 ± 0.74	3.58 ± 0.73	3.57 ± 0.76
Reliance on hunger and satiety cues	3.32 ± 0.98	3.32 ± 0.99	3.31 ± 0.90
Eating for physical rather than emotional reasons	3.12 ± 0.95	3.04 ± 0.95	3.51^*^ ± 0.87
Body-food choice congruence	3.32 ± 0.97	3.64 ± 0.99	3.67 ± 0.88
Three Factor Eating Questionnaire -Revised 21 (TFEQ- R21)			
Cognitive Restraint	41.62 ± 21.26	41.58 ± 10.60	41.86 ± 21.82
Uncontrolled eating	32.75 ± 20.24	32.81 ± 20.77	32.48 ± 17.52
Emotional eating	38.58 ± 27.50	40.73 ± 27.71	28.34^*^ ± 24.58

^*^Welch’s t test, p < .01

^a^Brazilian minimum wage was US$202.48 in 2020 and US$213.14 in 2021

### Associations of sex, BMI, age and Intuitive Eating with maladaptive eating behaviors

Men scored higher on total Intuitive Eating (mean = 3.49, SD = 0.57 vs. mean = 3.33, SD = 0.68, p < 0.01) and Eating for Physical Rather than Emotional Reasons (mean = 3.51, SD = 0.87 vs. mean = 3.04, SD = 0.95, p < 0.01) and lower on Emotional Eating compared to women (mean = 28.34, SD = 24.58 vs. mean = 40.73 SD = 27.71, p < 0.01). Spearman's correlations revealed several associations among the variables, as detailed in Table 2. Intuitive Eating presented a moderate negative relationship with BMI (rs =  − 0.39, p < 0.01), with similar results for the Eating for Physical Rather than Emotional Reasons and Reliance on Hunger and Satiety Cues subscales which also showed moderate negative correlations, (rs =  − 0.31, p < 0.01 and rs =  − 0.35, p < 0.01, respectively). Maladaptive eating behaviors, on the other hand, had positive associations with BMI, particularly Emotional Eating, with a moderate correlation (rs = 0.32, p < 0.01), and age exhibited a weak negative correlation with Unconditional Permission to Eat (rs =  − 0.07, p < 0.05), a weak positive correlation with Cognitive Restraint (rs = 0.11, p < 0.01) and no other significant associations.

**Table 2:** Spearman’s rho correlations for age, BMI, IES-2 and TFEQ-R21 total sample’ scores

Variable	1	2	3	4	5	6	7	8	9	10
1. Age	–									
2. BMI	.26**	–								
3. IES-2 total	− .02	− .39**	–							
4. IES-2 UPE	− .07*	− .25*	.65**	–						
5. IES-2 EPR	.02	− .31**	.83**	.40**	–					
6. IES-2 RHSC	− .04	− .35**	.77**	.42**	.43**	–				
7. IES-2 B-FCC	.02	− .18**	.49**	.06*	.33*	.36**	–			
8. TFEQ-R21 CR	.11**	.27**	− .31**	− .58**	− .11**	− .26**	.08**	–		
9. TFEQ-R21 UE	− .03	.29**	− .57**	− .30**	− .56**	− .42**	− .29**	.14**	–	
10. TFEQ-R21 EE	.00	.32**	− .73**	− .40**	− .84**	− .38**	− .28**	.16**	.62**	–

IES-2: Intuitive Eating Scale-2; UPE: unconditional permission to eat; RHSC: reliance on hunger and satiety cues; EPR: eating for physical rather than emotional reasons; B-FCC: body-food choice congruence; TFEQ-R21: Three-Factor Eating Questionnaire-Revised 21; CR: cognitive restraint; UE: uncontrolled eating; EE: emotional eating

^*^p < .05. ^**^p < .01

Intuitive Eating and its domains demonstrated a negative association with all maladaptive eating behaviors, showing moderate to strong negative associations predominantly. Total Intuitive Eating score exhibited a moderate negative correlation with Cognitive Restraint (rs =  − 0.31, p < 0.01), and strong negative correlations with Uncontrolled Eating (rs =  − 0.57, p < 0.01), and Emotional Eating (rs =  − 0.73, p < 0.01).

Upon analyzing subscales, Unconditional Permission to Eat showed a large negative correlation with Cognitive Restraint (rs =  − 0.58, p < 0.01). The Eating for Physical Rather than Emotional Reasons subscale exhibited the strongest negative correlations with Uncontrolled Eating (rs =  − 0.56, p < 0.01) and Emotional Eating (rs =  − 0.84, p < 0.01). Regarding the Reliance on Hunger and Satiety Cues subscale, moderate negative correlations were also observed with Uncontrolled Eating (rs =  − 0.42, p < 0.01) and Emotional Eating (rs =  − 0.38, p < 0.01). Unlike the other subscales, the correlation between Body-Food Choice Congruence with Cognitive Restraint was positive (rs = 0.08, p < 0.01).

In the MANCOVA model (see Table 3), all factors and covariates showed a significant multivariate effect on the combined dependent variables (p < 0.05), except for age. Eating for Physical Rather than Emotional Reasons and Unconditional Permission to Eat exhibited the largest multivariate effect sizes (Z = 603.97, η2p = 0.596; Z = 190.16, η2p = 0.317, respectively). In the univariate analyses, the model showed large effect sizes for all dependent variables, with large effects for Cognitive Restraint (η2p = 0.414), Uncontrolled Eating (η2p = 0.394), and Emotional Eating (η2p = 0.725). Unconditional Permission to Eat showed a large partial effect on Cognitive Restraint (η2p = 0.314), while Eating for Physical Rather than Emotional Reasons showed a large partial effect on Emotional Eating (η2p = 0.587).

**Table 3:** Effects of sex, BMI, age and IES-2 subscales on cognitive restraint, uncontrolled eating and emotional eating (n = 1237; MANCOVA)

Multivariate tests				
Effect	Pillai’s trace	Z	η^2^p	Observed power
Sex	.021	8.766^**^	.021	0.995
BMI	.048	20.608^**^	.048	1.000
Age	.003	1.182	.003	0.320
IES-2 UPE	.317	190.164^**^	.317	1.000
IES-2 RHSC	.055	23.793^**^	.055	1.000
IES-2 EPR	.596	603.966^**^	.596	1.000
IES-2 B-FCC	.025	10.610^**^	.025	0.999

Between subjects’ effects							
	TFEQ-R21 CR		TFEQ-R21 UE			TFEQ-R21 EE	
	Ze	η^2^p		Ze	η^2^p	Ze	η^2^p
Adjusted model	124.04^***^	.414^a^		114.33^***^	.394^b^	111.98^***^	.725^c^
Sex	5.18^**^	.004		8.98^***^	.007	4.75^**^	.004
BMI	41.85^***^	.033		8.82^***^	.007	20.08^***^	.016
Age	0.64	.001		1.91	.002	0.18	.000
IES-2 UPE	563.00^***^	.314		0.14	.000	12.42^***^	.010
IES-2 RHSC	10.16^***^	.008		52.82^***^	.041	0.00	.000
IES-2 EPR	45.79^***^	.036		261.85^***^	.176	1745.99^***^	.587
IES-2 B-FCC	26.17^***^	.021		5.25^**^	.004	0.80	.001

Parameter’s estimatives						
	TFEQ-R21 CR		TFEQ-R21 UE		TFEQ-R21 EE	
	β	η^2^p	β	η^2^p	β	η^2^p
Men	− 2.89	.004	3.69	.007	− 2.46	.004
BMI	41.49	.033	18.43	.007	25.56	.016
Age	3.45	.001	− 5.75	.002	1.63	.000
IES-2 UPE	− 17.26	.314	− .27	.010	− 2.28	.010
IES-2 RHSC	− 1.88	.008	− 4.15	.041	.042	.000
IES-2 EPR	4.15	.036	− 9.60	.176	− 22.78	.587
IES-2 B-FCC	2.74	.021	− 1.18	.004	− .42	.001

IES-2: Intuitive Eating Scale-2; UPE: unconditional permission to eat; RHSC: reliance on hunger and satiety cues; EPR: eating for physical rather than emotional reasons; B-FCC: body-food choice congruence; TFEQ-R21: Three Factor Eating Questionnaire-Revised 21; CR: cognitive restraint; UE: uncontrolled eating; EE: emotional eating

^*^p < .05. ^**^p < .01. ^***^p < .001. ^a^R^2^ = .414 (Adjusted R^2^ = .411); ^b^R^2^ = .394 (Adjusted R^2^ = .391); ^c^R^2^ = .725 (Adjusted R^2^ = .723)

In the parameter estimates, the largest partial effect on Cognitive Restraint was observed for Unconditional Permission to Eat (β = − 17.26, p < 0.001, η2p = 0.314). For Uncontrolled Eating, Eating for Physical Rather than Emotional Reasons demonstrated the largest partial effect (β = − 9.60, p < 0.001, η2p = 0.176). Regarding Emotional Eating, Eating for Physical Rather than Emotional Reasons showed the largest partial effect (β = − 22.78, p < 0.001, η2p = 0.587). Being male was also associated with lower Cognitive Restraint (β = − 2.98, p < 0.05, η2p = 0.001) and Emotional Eating (β = − 2.46, p < 0.05, η2p = 0.004), and higher Uncontrolled Eating (β = 3.69, p < 0.01, η2p = 0.007).

## Discussion

The present study aimed to identify the relationships between intuitive eating, its domains, and maladaptive eating behavior dimensions, namely cognitive restraint, uncontrolled eating, and emotional eating, while controlling for factors such as sex, BMI, and age. Our model significantly explained all dimensions of eating behavior. A negative association between intuitive eating and maladaptive eating behaviors was identified, with strong correlations observed for Uncontrolled Eating (rs =  − 0.57, p < 0.01) and Emotional Eating (rs =  − 0.73, p < 0.01), suggesting distinct patterns of relationship with food. In the MANCOVA model, a substantial proportion of variance was explained, particularly for Emotional Eating (η^2^p = 0.725).

Unconditional Permission to Eat was strongly and negatively associated with Cognitive Restraint (rs =  − 0.58, p < 0.01; β =  − 17.26, p < 0.001), while Eating for Physical rather than Emotional Reasons was negatively associated with both Uncontrolled Eating (rs =  − 0.56, p < 0.01; β =  − 9.6, p < 0.001) and Emotional Eating (rs =  − 0.84, p < 0.01; β =  − 22.78, p < 0.001). Although age showed correlations with Unconditional Permission to Eat and Cognitive Restraint, its effect was not significant in the MANCOVA analyses. Relationships between eating behavior and BMI were also observed, such as the negative association of Intuitive Eating with BMI (rs =  − 0.39, p < 0.01), particularly with domains related to internal regulation of eating, and the positive association between maladaptive eating behaviors and BMI, especially Emotional Eating (rs = 0.32, p < 0.01), indicating how they may influence food consumption and possible body weight gain.

In a Brazilian sample of 44 nutrition students, intuitive eating also showed strong negative correlations with restrictive eating and emotional eating [28]. Across a cross-country study involving samples of young adults from eight countries, similar associations were found, although with less strength due to sample heterogeneity [24]. In Christoph et al.'s [15] five-year longitudinal study, individuals with higher levels of intuitive eating engaged less in dieting or unhealthy weight control methods and experienced less binge-eating. This pattern is consistent with other cross-sectional and longitudinal studies, as summarized in a meta-analysis indicating that intuitive eating is negatively associated with restrictive eating, binge-purge eating, and, most strongly, emotional eating [23], which aligns with the strong negative correlation observed in the present study (rs =  − 0.73).

Some studies have identified sex differences in intuitive eating and maladaptive eating behaviors. In the cross-sectional study by Ayyıldız et al. [6], men exhibited higher levels of intuitive eating and its domains compared to women, while women reported higher levels of Emotional Eating. These findings are consistent with the present results and with previous studies [3]. Sociocultural pressures and the objectification of the female body have been suggested as key factors influencing these differences. For instance, Murray et al. [26] found that greater body vigilance and lower aesthetic satisfaction were associated with lower levels of intuitive eating and reduced trust in hunger and satiety signals, while greater investment in appearance was linked to lower Eating for Physical rather than Emotional Reasons in women. These findings align with the Acceptance Model of Intuitive Eating [4, 23], which proposes that body acceptance facilitates reliance on internal cues and resistance to external pressures. Accordingly, greater exposure to body-related pressures may impair women’s attunement to internal signals and increase vulnerability to emotional eating. This may also help explain lower levels of intuitive eating among individuals with higher body weight, who are more likely to experience weight stigma [11].

The relationship between intuitive eating and weight is already well-established in the literature [3, 6, 15, 23, 24]. However, in Brazil, there are still relatively few studies conducting such analyses. In dos Santos's study [19], students who gained weight during the Covid-19 pandemic exhibited lower levels of intuitive eating and its domains, except for unconditional permission to eat. What seems to play a greater role in the weight stability observed in intuitive eaters [34] is their attunement to interoceptive awareness, which leads them to trust and eat in accordance with their signals of self-regulation in eating, even in adverse situations. Additionally, this attunement likely influences emotional regulation, which when not developed adaptively with healthy coping strategies, among other factors, can increase susceptibility to emotional eating. Emotional eating, in turn, is associated with higher BMI and weight gain [35], which is consistent with the positive association observed in the present study (rs = 0.32).

There are still few intervention studies on intuitive eating, but available evidence shows promising trends. In a randomized clinical trial, participants reduced dietary restraint and increased intuitive eating, with maintenance over time [22]. Similarly, a systematic review found that all interventions increased intuitive eating and improved symptoms of disordered eating, with sustained effects in follow-up studies [7]. These findings are particularly relevant considering that Eating for Physical rather than Emotional Reasons showed the strongest association with Emotional Eating in the present study (β =  − 22.78). Notably, participants in an intervention study also reported increases in interoceptive awareness and self-compassion [13], suggesting potential mechanisms underlying these changes.

Interoceptive awareness, the ability to perceive and interpret internal bodily signals such as hunger and satiety [34], appears to play a central role in eating self-regulation. Greater attunement to these signals may promote eating in response to physiological needs rather than emotional triggers, which is consistent with the strong negative association observed in the present study between Eating for Physical rather than Emotional Reasons and Emotional Eating [34, 35]. From a clinical perspective, interventions targeting interoceptive awareness, such as mindfulness-based and body-oriented approaches, may represent promising strategies for reducing maladaptive eating behaviors and preventing disordered eating in university populations [7, 13, 22].

The strengths of this study include its national sample, with participants from all regions of the country, with approximately 48% of the sample coming from regions other than the Southern, rather than being solely regional like other Brazilian studies on the topic. We also highlight the utilization of validated instruments for collecting data on intuitive eating and behavior within the Brazilian population.

However, some limitations should be considered. Although participants were recruited nationwide, a high proportion of the sample was from the Southern region of Brazil, which may limit the representativeness of the findings. Also, detailed information regarding participants’ recruitment sources was not collected, which precluded the characterization of the sample according to recruitment platform and limited a more nuanced understanding of participants’ recruitment origins. Weight and height were self-reported and may be subject to systematic reporting errors, such as underreporting of weight and overreporting of height, potentially affecting the accuracy of BMI estimates.

Additionally, self-reported clinical diagnoses, including eating disorders, may be subject to underreporting or misclassification. The cross-sectional design does not allow for causal inference. Moreover, a broader range of eating behaviors was not assessed, including aspects such as emotional undereating or eating in response to positive emotions, which may further contribute to understanding the complexity of eating patterns. Future studies should aim to recruit more geographically balanced samples, collect detailed information on recruitment channels, and, where feasible, incorporate objective anthropometric measurements and clinically validated assessments of eating disorders.

## Conclusions

In conclusion, among Brazilian university students, intuitive eating is negatively associated with cognitive restraint, uncontrolled eating, and emotional eating. The aspects of intuitive eating most strongly linked to these behaviors are eating for physical rather than emotional reasons and unconditional permission to eat, highlighting their potential relevance as targets for interventions aimed at improving eating behaviors.

## What is already known on this subject?

Intuitive eating is negatively associated with BMI, linked to weight stability, as well as improved psychosocial health, less disordered eating, and greater body appreciation.

## What does this study add?

This study presents data on intuitive eating and eating behavior from a national Brazilian sample. The findings highlight the importance of developing interventions that help individuals cultivate awareness, learn to distinguish between emotional and physiological hunger cues, and build trust in their body's signals to guide eating behavior. Additionally, these interventions should promote healthy emotional regulation strategies, taking gender differences into account, with the goal of reducing restrictive, uncontrolled, and emotional eating.

## Data Availability

The data that support the findings of this study are available from the authors upon reasonable request.
